# Low Frequency of Acquired Isoniazid and Rifampicin Resistance in Rifampicin-Susceptible Pulmonary Tuberculosis in a Setting of High HIV-1 Infection and Tuberculosis Coprevalence

**DOI:** 10.1093/infdis/jix337

**Published:** 2017-07-20

**Authors:** Neesha Rockwood, Frederick Sirgel, Elizabeth Streicher, Robin Warren, Graeme Meintjes, Robert J Wilkinson

**Affiliations:** 1 Department of Medicine, Imperial College, and; 2 Francis Crick Institute, London,United Kingdom; and; 3 Wellcome Centre for Infectious Diseases Research in Africa, Institute of Infectious Disease and Molecular Medicine, University of Cape Town, Cape Town, and; 4 Faculty of Health Sciences, Stellenbosch University, Tygerberg, South Africa

**Keywords:** Acquired/amplified drug resistance, *Mycobacterium tuberculosis*, HIV-1 coinfection, tuberculosis treatment outcomes, isoniazid monoresistance, minimum inhibitory concentrations

## Abstract

**Background:**

We estimated the incidence of acquired isoniazid and rifampicin resistance in rifampicin-susceptible tuberculosis in a setting of high human immunodeficiency virus type 1 (HIV-1) infection and tuberculosis coprevalence.

**Methods:**

GeneXpert MTB/RIF–confirmed patients with rifampicin-susceptible tuberculosis were recruited at antituberculosis treatment initiation in Khayelitsha, South Africa. Liquid culture and adherence assessment were performed at 2 and 5–6 months. MTBDRplus was performed on mycobacteria-positive cultures to ascertain acquired drug resistance (ADR). Spoligotyping and whole-genome sequencing were performed to ascertain homogeneity between baseline isolates and isolates with ADR. Baseline isolates were retrospectively tested for isoniazid monoresistance. An electronic database review was performed to ascertain tuberculosis recurrences.

**Results:**

A total of 306 participants (62% with HIV-1 coinfection, of whom 71% received antiretroviral therapy) were recruited. Ascertainment of outcomes was complete for 284 participants. Five acquired a resistant *Mycobacterium tuberculosis* strain during or subsequent to treatment. One strain was confirmed to have ADR, 2 were confirmed as causing exogenous reinfection, and 2 were unrecoverable for genotyping. Incident ADR was estimated to have ranged from 0.3% (95% confidence interval [CI], .1%–1.9%; 1 of 284 participants) to 1% (95% CI, .2%–3%; 3 of 284 participants). Seventeen of 279 baseline isolates (6.1%; 95% CI, 3.6%–9.6%) had isoniazid monoresistance (13 of 17 had an *inhA* promoter mutation), but 0 of 17 had amplified resistance.

**Conclusions:**

Treatment with standardized antituberculosis regimens dosed daily throughout, high uptake of antiretroviral therapy, and low prevalence of isoniazid monoresistance were associated with a low frequency of ADR.

## Summary

In a setting of high tuberculosis and human immunodeficiency virus type 1 infection coprevalence, low prevalence of isoniazid monoresistance, treatment with daily standardized antituberculosis regimens throughout and high uptake of antiretroviral therapy were associated with low frequency of acquired drug resistance. Prevention of transmitted resistance should be prioritized.

In a setting of high tuberculosis and human immunodeficiency virus type 1 infection coprevalence, low prevalence of isoniazid monoresistance, daily treatment with standardized antituberculosis regimens throughout the study period, and high uptake of antiretroviral therapy were associated with low frequency of acquired drug resistance. Prevention of transmitted resistance should be prioritized.

Tuberculosis remains the foremost cause of death in human immunodeficiency virus type 1 (HIV-1)–infected individuals in Africa. An increasing problem is the acquisition and transmission of drug-resistant strains of *Mycobacterium tuberculosi*s. In 2015, in South Africa there were 19613 laboratory-diagnosed cases of rifampicin (RIF) resistance among an estimated 454000 incident tuberculosis cases [1].

Acquired drug resistance (ADR) is the amplification and fixation of both new mutations and minority preexisting mutants within a clonal population, leading to phenotypic resistance. This process occurs during drug treatment. The transmission of drug-resistant *M. tuberculosis* strains to newly infected individuals is known as transmitted drug resistance. Our recent systematic review showed a significantly increased risk of acquired RIF and/or isoniazid (INH) resistance with baseline monoresistance/polyresistance and with HIV-1 coinfection [2]. However, HIV-1 infection was not an increased risk factor for ADR in African countries with a high coprevalence of HIV infection and tuberculosis. With earlier commencement of antiretroviral therapy (ART), as per programmatic guidelines, it remains to be seen whether HIV-1 coinfection will continue to be associated with ADR. A study conducted in India showed that, in patients receiving a thrice-weekly tuberculosis regimen, ART reduced but did not eliminate the risk of ADR [3]. Incident ADR cases are hypothesized to fuel and sustain transmission of resistance. In many resource-limited settings, including the South African tuberculosis program, the GeneXpert MTB/RIF test is used for baseline RIF resistance. Baseline INH monoresistance is largely undetected. According to programmatic guidelines, treatment for those who are smear negative at 2 months should be switched to the continuation-phase therapy with RIF/INH. Hence, individuals with INH monoresistance will effectively receive RIF monotherapy, and there is potential for amplification of drug resistance.

We performed a prospective cohort study to determine the incidence of and risk factors for ADR in Khayelitsha, Western Cape, South Africa. We aimed to determine the proportion of tuberculosis cases with RIF susceptibility at baseline that go on to acquire drug resistance, allowing us to indirectly estimate the contribution of ADR to the drug-resistant tuberculosis epidemic.

## METHODS

### Setting

Khayelitsha is a predominantly black African township, with a population of approximately 400000. There are high rates of unemployment, overcrowding, and households in informal dwellings. The burden of HIV-1 infection is extremely high (antenatal prevalence, 37%). There were 4695 registered tuberculosis cases in 2014 (54% were microbiologically confirmed) and 227 cases of RIF-monoresistant tuberculosis or multidrug-resistant tuberculosis (MDR-TB; routine tuberculosis data, City Health).

Participant recruitment was at Site B Ubuntu Clinic, a primary care integrated HIV/tuberculosis clinic, during March 2013–July 2014. Patients with GeneXpert MTB/RIF-confirmed RIF-susceptible pulmonary tuberculosis were recruited at the commencement of tuberculosis therapy. Exclusion criteria included age of <18 years, receipt of treatment for tuberculosis within the previous 6 months, a positive result of a pregnancy test, decline of HIV testing, inability to expectorate sputum, or receipt of ≥3 doses of tuberculosis treatment before screening. Patients received routine programmatic management via the Ubuntu Clinic. Patients received directly observed treatment during the first 1–2 weeks, during which time they received adherence counseling and a home visit. Thereafter, the majority received a monthly supply of antituberculosis drugs, which were self-administered. The intensive phase of treatment consisted of daily RIF/INH/pyrazinamide (PZA)/ethambutol (EMB) therapy for 2 months, and the continuation phase involved daily RIF/INH therapy for 4 months (2[RIF/INH/PZA/EMB]_7_ 4[RIF/INH]_7_) [4]. The switch from the intensive phase to the continuation phase was guided by smear conversion after 2 months for those who were smear positive at baseline. The study received ethics approval from the University of Cape Town Human Research Ethics Committee (reference 568/2012).

### Procedures and Outcomes

Data were collected on sociodemographic characteristics, contacts with MDR-TB, previous tuberculosis treatment, and comorbidities. Participants underwent sputum induction (using 3% saline). Baseline bacterial load was estimated via smear grading and days to culture positivity in liquid mycobacterial growth indicator tubes (MGITs). The presence of cavitation with a >1-cm maximum diameter on a chest radiograph was noted. Extensive disease on a chest radiograph was noted as either involvement of >1 lung lobe or involvement of ≥1 of 3 (upper, middle, or lower) zones per lung. Participants underwent HIV testing (by serologic analysis), determination of CD4^+^ T-lymphocyte count, and quantification of the HIV-1 load. At 2 months and 5–6 months, adherence was reviewed via 2 different methods: (1) missing ≥5 doses in the previous month, based on pill counts and/or self-report on interview; and (2) use of the Arkansas test to evaluate an archived 2-month urine specimen [5].

At the 2-month and 5–6-month follow-up visits, participants underwent sputum induction with nebulized 3% saline. Sputum cultures yielding mycobacteria were screened for ADR to RIF and INH, using the GenoType MTBDRplus line probe assay, version 2.0 (Hain Lifescience, Nehren, Germany).

Spoligotyping (Mapmygenome.in, Hyderabad, India) was performed by the internationally standardized method [6] on sequential isolates from cases with new resistance detected, to investigate dual mixed infection at baseline and exogenous reinfection during treatment. Where the spoligotype was identical, we performed whole-genome sequencing (WGS) to further assess sublineage variation [7].

Participants were reviewed at 5–6 months to ascertain treatment outcome as per the World Health Organization definition [8]. Electronic database searches were conducted of the Western Cape Department of Health Data Repository and the National Health Laboratory Service database to ascertain reported deaths and tuberculosis recurrences until November 2015. As per national program guidelines, sputum specimens from identified cases underwent smear grading and analysis by the GeneXpert MTB/RIF test, and if RIF resistance suspected, phenotypic drug susceptibility testing (DST)/line probe assay performed on a separate sputum specimen sent for culture. Hence, unless the patients died prematurely, laboratory ascertainment of DST results was complete in the majority of cases. “True recurrence” was defined as culture positivity and/or smear 2+/3+ positivity. “Possible recurrence” was defined as positive GeneXpert MTB/RIF result with a resistance profile differing from that at baseline and/or a smear grading of scanty/1+ positivity in the absence of culture confirmation.

We retrospectively tested for INH monoresistance in 279 stored baseline isolates through use of the MTBDRplus test and/or minimum inhibitory concentration (MIC) analysis. Clinical notes were reviewed to determine the duration of treatment and outcomes in cases of baseline INH monoresistance. MICs were determined for isolates from a randomly selected subcohort of 109 participants at baseline and from the first 20 consecutive participants with paired cultures at baseline and 2 months, using the Bactec MGIT 960 system with EpiCenter software and the TBeXist application (Supplementary Methods) [9]. MIC analyses were performed in triplicate. As a difference of 1 log_2_ between isolates could be attributed to technical error, a significant change in MIC in paired baseline and 2-month cultures was predefined as a ≥4-fold increase or decrease. For participants with ADR, extended phenotypic DST was performed using Sensititre MycoTB plates (Trek Diagnostic Systems) as previously described (Supplementary Table 1) [10].

### Statistical Analyses

If a 1% incidence of ADR is assumed, 299 patients must be followed up during chemotherapy to have a 95% probability of observing at least 1 case of ADR. The proportion of participants with ADR was calculated, inclusive of the whole cohort and restricted to those in whom ascertainment was complete. All figures and analyses were performed in GraphPad (La Jolla, CA) Prism.

## RESULTS

### Clinical Characteristics of the Cohort

**Figure 1.**
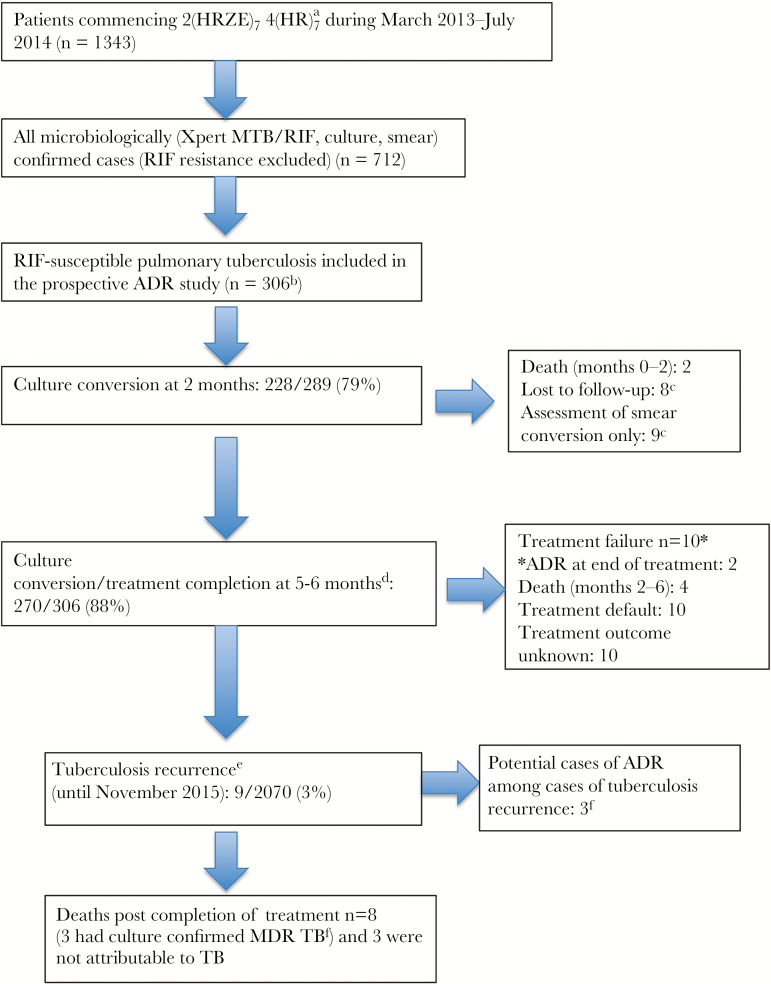
Study recruitment and participant outcomes. ^a^The regimen consisted of a 2-month initial phase of a daily fixed-dose combination of isoniazid, rifampicin (RIF), pyrazinamide, and ethambutol, following by a 4-month continuation phase of a daily fixed-dose combination of isoniazid and RIF. ^b^Reasons why patients were not recruited to the acquired drug resistance (ADR) study were as follows: (1) they had received ≥3 doses of tuberculosis treatment (includes individuals were transferred in from other clinics/hospitals and those who started treatment on days the study team was not recruiting), (2) they declined participation or were unable to provide informed consent, (3) they were unwilling to undergo human immunodeficiency virus (HIV) testing, and (4) they were unable to expectorate sputum. ^c^Participants were not included in the denominator. ^d^A total of 240 participants had assessment of culture conversion at the end of treatment. A further 40 were assessed as treatment completers or underwent assessment of smear conversion. ^e^Eight recurrences were either culture confirmed and/or smear positive (grading, 2+/3+ [definite recurrence]). One of these recurrences was symptomatic, scanty smear positive, and had a confirmatory Xpert MTB/RIF test revealing RIF susceptibility (possible recurrence). ^f^Findings are for the same 3 individuals. Abbreviation: MDR, multidrug resistant.

A total of 306 patients with microbiologically confirmed RIF-susceptible pulmonary tuberculosis were recruited. Figure 1 outlines study recruitment in the context of the overall clinic tuberculosis treatment initiators. Patients in the cohort had clinical follow-up for the duration of tuberculosis treatment. They had a median of 22 months (interquartile range [IQR], 18–28 months) follow-up, beginning at commencement of tuberculosis treatment, for ascertainment of disease recurrence via electronic database searches.

Demographic and clinical characteristics were comparable between the study cohort and the overall clinic (Table 1). None of the study participants had known contacts with MDR-TB or had received previous INH preventive therapy. Of note was the observation that 57 of 180 male participants (32%) had previously been in prison. A total of 191 of 306 participants (62%) were coinfected with HIV-1. The median CD4^+^ T-lymphocyte count was 231 cells/mm^3^ (IQR, 101–376 cells/mm^3^), and 47 of 191 (25%) had a CD4^+^ T-cell count of <100 cells/mm^3^. Seventy-four of 191 (39%) were receiving ART at baseline, and 47 of 191 (25%) had achieved virologic suppression. Twenty of 59 patients (34%) who had received ART for >6 months at baseline had a viral load of >200 copies/mL. By 2 months, 135 of 191 HIV-1–coinfected patients (71%) were receiving ART. Table 1 details clinical characteristics of the study cohort, stratified by HIV-1 serostatus. The proportion of the cohort considered to be nonadherent at the 2/5–6-month reviews was 24 of 284 (8%) according to pill count or self-report and 36 of 285 (13%) according to the Arkansas test.

**Table 1.** Characteristics of the Study Cohort and Overall Clinic Population, by Human Immunodeficiency Virus Type 1 (HIV-1) Infection Status

Characteristic	Study Cohort (n = 306)^a^		Overall Clinic Population^a,b^ (n = 712)	
	HIV-1 Infected (n = 191)	HIV-1 Uninfected (n = 115)	HIV-1 Infected (n = 448)	HIV-1 Uninfected (n = 252)
Male sex	48	77	47	70
Retreated	34	30	32	29
Age, y	35 (30–42)	34 (27–49)	36 (31–43)	37 (29–52)
Xhosa ethnicity	95	99	NA	NA
Smoking status				
Never	57	43	NA	NA
Former	22	20	NA	NA
Current	21	37	NA	NA
Alcohol use	33	43	NA	NA
Recreational drug use	5	7	NA	NA
Former prisoner	20	20	NA	NA
Former miner	4	10	NA	NA
Diabetic	5	7	NA	NA
Drug side effects during treatment	36	25	NA	NA
BMI^c^	22 (20–24)	20 (19–23)	NA	NA
Extensive radiological disease	55	83	NA	NA
Cavitations	35	53	NA	NA
CD4^+^ T-cell count, cells/mm^3^	231 (101–376)	…	210 (96–357)	…
VL <40 copies/mL at baseline	25	…	NA	…
Receiving ART at baseline	39	…	34	…
Smear positive^d^	43	63	52	59
Time to culture positivity, d	12 (8–17)	9 (7–13)	NA	NA
Duration of intensive phase, wk, median	8	8	NA	NA
Total duration of treatment, mo, median	6	6	NA	NA

Data are percentage of patients or median value (interquartile range), unless otherwise indicated.

Abbreviations: ART, antiretroviral therapy; d, days; mo, months; NA, not available; VL, viral load; y, years.

^a^A total of 62% of the study cohort and 64% of the overall clinic population were infected with HIV-1; 38% and 36%, respectively, were not infected with HIV-1.

^b^Twelve cases with unknown status were excluded from overall clinic statistics.

^c^Body mass index (BMI) is calculated as the weight in kilograms divided by the height in meters squared.

^d^Defined as 1+ to 3+ on smear grading.

### Treatment Outcomes

By 2 months after treatment initiation, 228 of 289 participants (79%; 95% confidence interval [CI], 74%–83%) had experienced culture conversion. Two hundred seventy of 306 (88%; 95% CI, 84%–92%) had an outcome of cure or treatment completion. There were 22 patients in whom ascertainment of ADR was incomplete (2 died before expectoration of sputum, 10 experienced treatment default, and 10 had an unknown treatment outcome; Supplementary Table 2). There were 10 treatment failures and 9 recurrences over the study follow-up period. There were 6 deaths during treatment and 8 subsequent to treatment completion, of which 5 were attributable to tuberculosis (Figure 1). Supplementary Table 3 shows treatment outcomes stratified by HIV-1 serostatus. There were 2 cases of new drug resistance identified during treatment. Their sequential phenotypic DST profiles and spoligotypes are outlined in Table 2. Both were smear negative and had cavitary disease detected by chest radiography at baseline and documented poor adherence to treatment. Case 1 (uninfected with HIV-1) was a retreatment case and had PZA monoresistance at baseline. Case 2 was a new tuberculosis case and had recently received diagnoses of both HIV-1 coinfection and type 2 diabetes mellitus. Case 2 was subsequently reclassified as exogenous reinfection, based on sublineage typing via WGS.

**Table 2.** Cases of New Drug Resistance During Treatment

Case, Time Point	Regimen	International Spoligotype Family^a^	MIC, µg/mL						
			**Sensititre MycoTB Plate Method**			**Bactec 960 Method**			
			INH (CC, 0.2/1 μg/mL)	RIF (CC, 1 μg/mL)	EMB (CC, 5/10 μg/mL)	PZA (CC, 100 μg/mL)	INH (CC, 0.1 μg/mL)	RIF (CC, 1 μg/mL)	EMB (CC, 5 μg/mL)
Case 1									
Baseline	RIF/INH/PZA/EMB	LAM3	0.06–0.12 (S)	0.12 (S)	2 (S)	>100 (R)	ND	ND	ND
Month 2^a^	RIF/INH/PZA/EMB	LAM3	2 (R)^c^	0.12(S)	1 (S)	>100 (R)	0.05 (S)^c^	ND	ND
Month 5	RIF/INH	LAM3	2 (R)	0.12 (S)	1 (S)	>100 (R)	ND	ND	ND
Month 7	RIF/INH	LAM3	2 (R)	0.12 (S)	1 (S)	>100 (R)	ND	ND	ND
Case 2									
Baseline	RIF/INH/PZA/EMB	Beijing	0.03 (S)	0.12 (S)	1.0 (S)	≤100 (S)	0.05 (S)	0.125 (S)	≤2.5 (S)
Month 2	RIF/INH/PZA/EMB	Beijing	0.03 (S)	0.12 (S)	1.0 (S)	≤100 (S)	0.05 (S)	0.125 (S)	≤2.5 (S)
Month 5^b^	RIF/INH	Beijing	0.06 (S)	>16 (R)	2.0 (S)^c^	>100 (R)	>1, ≤10 (R)^c^	>20 (R)	10 (R)^c^
Month 7	RIF/INH	Beijing	2 (R)	>16 (R)	4–8 (S)^c^	>100 (R)	>1, ≤10 (R)^c^	>20 (R)	10 (R)^c^

Abbreviations: CC, critical concentration; EMB, ethambutol; INH, isoniazid; ND not done; PZA, pyrazinamide; R, resistant; RIF, rifampicin; S, susceptible.

^a^According to the SITVIT database.

^b^ *KatG* mutation S315T was only detected at 5 and 7 months by the MTBDRplus assay.

^c^ *rpoB* mutation S531L was detected at 5 and 7 months by the MTBDRplus assay. However, no *KatG* or *inhA* promoter mutation was detected.

^^d^^Discrepancy in drug susceptibility test result between the Sensititre MycoTB and MGIT 960 method.

Following treatment cure, 3 cases received a diagnosis of culture-confirmed drug-resistant tuberculosis (MDR in 2 cases and RIF monoresistance in 1 case) within 14 months of treatment cessation. All 3 cases died (Figure 1) and had advanced HIV disease (CD4^+^ T-lymphocyte count, <100 cells/mm^3^) at the time of death. The spoligotype of sequential isolates was different (LAM3/Beijing) in 1 of these 3 cases. The drug-resistant strain could not be recovered for spoligotyping in the other 2 cases. If it is assumed that the 2 recurrences were not exogenous reinfection, the proportion of cases with ADR in the cohort was 3 of 306 (1%; 95% CI, .2%–2.8%). The proportion of cases with ADR, excluding 22 for whom follow-up was incomplete, was 3 of 284 (1%; 95% CI, .2%–3.1%). On the assumption that the 2 recurrences were exogenous reinfections, the proportion of cases with ADR in the cohort was 1 of 284 (0.3%; 95% CI, .01%–1.9%).

### MIC Profiles

**Figure 2.**
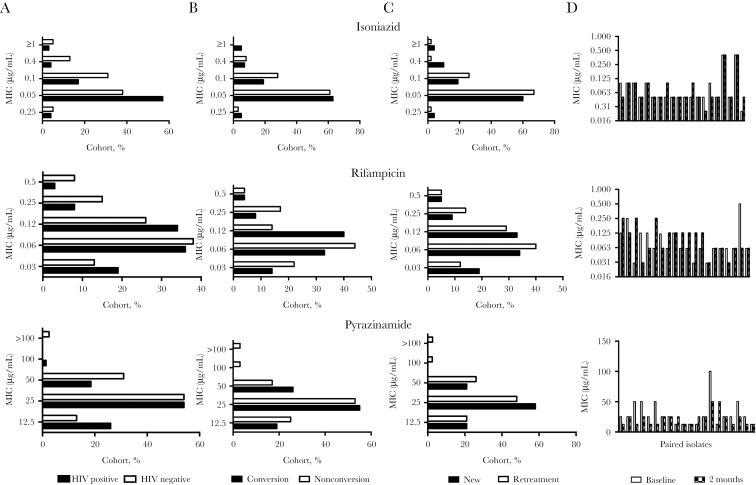
Minimum inhibitory concentration (MIC) distribution for rifampicin, isoniazid, and pyrazinamide. *A*–*C*, MICs in a subcohort of 109 *Mycobacterium tuberculosis* strains stratified by human immunodeficiency virus type 1 (HIV-1) infection status (*A*), 2-month culture conversion status (*B*), and retreatment status (*C*). *D*, MIC testing was performed on paired isolates (obtained at baseline and 2 months after treatment initiation) from 20 patients without culture conversion at 2 months and no genotypic evidence of acquired drug resistance.

As illustrated in Figure 2, the MIC profile at baseline, stratified by HIV-1 serostatus, retreatment status, and culture conversion status, were comparable. The median MICs for RIF, INH, and PZA were 0.06 μg/mL, 0.05 μg/mL, and 25 μg/mL. In the subset sampled, PZA resistance at baseline was 1 of 109 (0.1%). In a small cohort of 20 patients, the baseline MIC was compared to the MIC at 2 months. As illustrated by the paired plots in Figure 2, no significant increase in MIC occurred during treatment.

### Baseline INH Monoresistance

The baseline prevalence of INH monoresistance, determined by either MIC analysis and/or MTBDRplus testing, was 17 of 279 (6.1%; 95% CI, 3.6%–9.6%). Of 47 cases in which INH DST was performed by both methods, discordant results were observed for 3 (6.1%; Supplementary Figure 1). Forty-one percent of patients were HIV-1 coinfected, 18% were undergoing retreatment, and 12% had been previously in prison. All cases determined to have INH monoresistance by MTBDRplus had *inhA* promoter mutations. Treatment outcomes are shown in Table 3. There were no cases of treatment failure, relapse, or ADR (0%; 95% CI, 0%–19.5%). Although INH DST results were not available to program physicians and nurses in real time, the median duration of the intensive phase was >8 weeks (10.3 weeks; IQR, 8.4–12.6 weeks). Hence, clinical improvement, with or without radiological improvement, may have been assessed to be slower, necessitating a longer duration of the intensive phase. Of note, 15 of 17 participants were smear negative at 2 months and were thus eligible as per program guidelines [4] to be switched to the continuation phase. In no case was treatment intensified with additional antituberculosis drugs

**Table 3.** Characteristics and Outcomes at 2 Months and the End of Treatment Among Patients With and Those Without Isoniazid Monoresistance (IMR) Detected by Minimum Inhibitory Concentration Testing and/or the MTBDR *plus* Assay

Characteristic	IMR (n = 17)	No IMR (n = 262)
Male sex	59	59
HIV-1 infected	41	63
Retreatment	18	32
Age, y	36 (32–43)	34 (29–42)
BMI^a^	21 (19–23)	21 (19–24)
Former prisoner	12	20
Cavitations	41	43
Smear positive^b^	82	53
Duration of intensive phase, wk, median	10.3	8
Total duration of treatment, mo, median	6.3	6
Outcome		
At 2 mo		
Culture conversion, no. (%)	13 (76)	191^c^ (77^d^)
At end of treatment		
Successful		
Overall, no. (%)	16 (94)	227 (87)
Cure, no.	15	192
Treatment completion, no.	1	35
Unsuccessful, no.		
Overall	1	35
Loss to follow-up	1^e^	9
Treatment failure	0	10
Treatment default	0	10
Death	0	6

Data are percentage of patients or median value (interquartile range), unless otherwise indicated.

Abbreviation: HIV-1, human immunodeficiency virus type 1; mo, months; wk, weeks; y, years.

^a^Body mass index (BMI) is calculated as the weight in kilograms divided by the height in meters squared.

^b^Defined as 1+ to 3+ on smear grading.

^c^Two participants died before the 2-mo visit and were assumed not to have experienced culture conversion.

^d^The denominator used to calculate this percentage excludes 15 participants who did not produce sputum for culture at this time point.

^e^The participant was alive but refused to attend clinic for the end of treatment visit.

## DISCUSSION

In the current era of both timely ART and a daily tuberculosis treatment regimen consisting of 2(RIF/INH/PZA/EMB)_7_ 4(RIF/INH)_7_, there are limited data available on the frequency of and associated factors in the development of ADR in RIF-susceptible tuberculosis. Within this cohort, which had a baseline INH monoresistance of 6.1% (low-level resistance associated with *inhA*) and a significant proportion with advanced immunosuppression (25% with HIV-1 and tuberculosis and a CD4^+^ T-cell count of <100 cells/mm^3^), the estimated incidence of ADR was 0.3%–1%. Our data suggest transmitted drug resistance as the predominant perpetuator of the MDR-TB epidemic in Khayelitsha. This is supported by molecular epidemiology studies that have shown significant clustering of MDR *M. tuberculosis* strains in difference provinces of South Africa, implying ongoing transmission [11].

The generalizability of our findings of low rates of ADR to areas with elevated PZA/EMB resistance is unclear. Although PZA monoresistance is likely to have been a risk factor for acquired INH resistance in case 1 (Table 2), we observed that drug resistance was not amplified in patients with undiagnosed baseline INH monoresistance and that none experienced treatment failure or recurrence of tuberculosis despite receiving the standard treatment, which is prescribed for RIF-susceptible tuberculosis on the basis of a baseline GeneXpert MTB/RIF result. Given the small sample size, these results must be interpreted with caution. A recent meta-analysis showed treatment failure, tuberculosis relapse, and acquired MDR-TB in 11%, 10%, and 8% of patients, respectively [12]. Contrasting reports in the literature regarding the impact of INH monoresistance on treatment outcomes suggest that the proportion of patients requiring retreatment [13–16], the proportion with HIV-1 coinfection [3], and the frequency of dosing, number of efficacious drugs given, and duration of treatment [15, 17] determine treatment outcomes in patients with INH monoresistance. Factors which may have contributed to a favourable outcome in this cohort include, the majority of patients with INH monoresistance were new cases of tuberculosis and had low-level INH resistance causing mutations (in the *inhA* promoter; MIC range, 0.2–1 mg/L [18]). All received a daily regimen and had good adherence.

There was evidence suggestive of incomplete adherence to both ART (34% did not have virologic suppression at baseline) and tuberculosis treatment (8%–13%). No patient with good adherence developed ADR during treatment. Several previous studies have shown that nonadherence is a risk factor for ADR [19–21]. It is possible that we underestimated the occurrence of ADR among participants who experienced treatment default. A hypothetical increase in ADR from 1% to 2%–3% would influence both treatment outcomes and potentially increase transmitted drug resistance in cases of delayed diagnosis of ADR. In this cohort, 32% of men had previously spent time in prison. Poor health systems and fear of stigmatization are likely to contribute to unplanned treatment interruptions of both ART and tuberculosis treatment during incarceration [22].

Although pharmacokinetic variability may be a determinant of long-term outcomes, including ADR, we have previously shown in a subset of this cohort that the proportion with low concentrations of RIF, INH, and PZA was not significantly different between patients who experienced treatment failure or tuberculosis relapse and those with good long-term outcomes [23].

The MIC profiles of *M. tuberculosis* strains from a population enriched for HIV- infected participants were similar to MIC distributions from different geographical populations and lineages [24, 25]. Khayelitsha has a preponderance of *M. tuberculosis* strains of lineage 2 and 4 [26]. Retreatment status was not associated with higher MICs at baseline. Potential mechanisms of mycobacterial persistence and phenotypic tolerance are via altered transcriptional modification (with or without pretranscriptional or posttranscriptional modification), which allows subpopulations to adapt metabolically [27] to sterilizing drugs such as RIF and PZA, often within different microenvironments [28]. However, there was no evidence of an increase in MIC accumulated over the first 2 months of tuberculosis treatment in a comparison of paired isolates from baseline and 2 months after treatment initiation. Nor was there evidence of higher MICs for the baseline strains from patient who were still culture positive at 2 months.

There is significant variation in estimates of the incidence of ADR among cases of RIF-susceptible tuberculosis in the literature [2], which may be secondary to differences in study methods. Different methods may yield discrepant DST results for first-line tuberculosis drugs. This was seen in case 2 (Table 3), for whom MTBDRplus did not identify INH resistance, although INH resistance was phenotypically detected at both 5 and 7 months. PCR and probe-based molecular genotyping assays do not include all rare or “disputed” mutations associated with intermediate MICs [29]. In case 2, there was a discrepancy in EMB phenotypic susceptibility between the BACTEC 960 method and Sensititre MycoTB plate method at months 5 and 7. Certain phenotypic DST methods have also been shown to miss clinically relevant drug resistant mutations for both EMB and RIF [30–33]. Hence, assigning a gold standard to detect ADR is challenging and is a current field of research. The proportion method of phenotypic DST assigns growth of ≥1% at a predefined critical concentration as resistance [34]. After sputum decontamination, there are subculture stages and subsequent standardization of inoculum size as per MIC testing protocols. Hence, the bacilli in culture may not fully represent drug-resistant subpopulations in the original sputum specimen. Recent advances using WGS of sputum samples may help overcome this issue [35].

Confirmation that drug resistance was acquired/amplified during treatment requires ruling out infection at baseline due to 2 strains with discordant drug susceptibility (dual mixed infection) and subsequent exogenous reinfection. Spoligotyping was performed on sequential isolates to screen for the above. Conventional molecular typing, which uses mobile or repetitive elements (*IS6110* RFLP, spoligotyping, and MIRU-VNTR) lack discriminatory power [36, 37]. Through high-coverage WGS, strains with apparently identical fingerprints but discordant DST patterns have been shown to have significant diversity, with up to 130 single-nucleotide polymorphism differences [36]. Sublineage typing through WGS has enabled greater discriminatory power [7] and reclassification of case 2 (Table 2) as having exogenous reinfection.

There were several limitations in this study. Although rates of declined consent to participate in the study were low (<5%), we cannot rule out potential selection bias. The predominant cause of missed enrollment was previous commencement of treatment in other clinics or inpatient settings and during periods when the research team was unable to recruit (as the study progressed, we excluded all patients who had received >1 dose of tuberculosis treatment). However, baseline demographic characteristics, HIV-1 seropositivity, median CD4^+^ T-lymphocyte count, the percentage receiving ART at baseline, and the percentage with smear positivity in the study cohort and the overall clinic population were well matched (Table 1). However, it is possible that the patients included in the study were different from those in other clinics with respect to other unmeasured characteristics. As the program only ascertained smear conversion at 5–6 months, we could not compare treatment outcomes between the study cohort and the overall clinic by using culture conversion at the end of treatment. In our electronic database searches, we relied on patients actively presenting for diagnostic evaluation of their recurring symptoms/signs, and hence tuberculosis in a proportion of patients may have relapsed but remained undiagnosed in the community. In retrospectively ascertaining baseline INH monoresistance, not all isolates underwent both MIC analysis and MTBDRplus testing. Our analysis of outcomes in patients with baseline INH monoresistance and paired MIC analysis during treatment was limited by low numbers.

To summarize, in this setting with a high coprevalence of HIV infection and tuberculosis and a low prevalence of INH-monoresistant tuberculosis, treatment of RIF-resistant tuberculosis with a standardized tuberculosis regimen administered daily in both the intensive and continuation phases and high ART coverage were associated with a low frequency of acquired resistance to INH and RIF. Public health efforts should therefore emphasize prevention of onward transmission of drug resistance.

## Supplementary Data

Supplementary materials are available at *The Journal of Infectious Diseases* online. Consisting of data provided by the authors to benefit the reader, the posted materials are not copyedited and are the sole responsibility of the authors, so questions or comments should be addressed to the corresponding author.

## Supplementary Material

Supplementary_Table_1

Supplementary_Table_2

Supplementary_Table_3

Supplementary_Figure_1

Supplementary_methods

## Notes

***Acknowledgments.*** We thank Rene Goliath, Lebo Tsekela, Nonceba Gobe, Amanda Jackson, Patrick Hamadi, and Vanessa January of the Wellcome Centre for Infectious Diseases Research in Africa and the clinical and administrative staff of the Western Cape Department of Health.

***Financial support.*** This work was supported by the Wellcome Trust (grants WT 104803, WT203135 [to R. J. W.], and WT 098316 [to G. M.]); the Francis Crick Institute, which receives its core funding from Cancer Research UK, the UK Medical Research Council, and the Wellcome Trust (grant 00110218 to R. J. W.); the European Union (grant EU FP7 HEALTH-F3-2012–305578 to R. J. W.); the National Research Foundation of South Africa (grant 96841 to R. J. W. and grant 64787 and NRF incentive funding [UID 85858] to G. M.); and the South African Medical Research Council, through its TB and HIV Collaborating Centres Programme, with funds received from the National Department of Health (grant RFA# SAMRC-RFA-CC: TB/HIV/AIDS-01-2014 to G. M.).

***Potential conflicts of interest.*** All authors: No reported conflicts of interest. All authors have submitted the ICMJE Form for Disclosure of Potential Conflicts of Interest. Conflicts that the editors consider relevant to the content of the manuscript have been disclosed.
