# Supplementary material for: Low Frequency of Acquired Isoniazid and Rifampicin Resistance in Rifampicin-Susceptible Pulmonary Tuberculosis in a Setting of High HIV-1 Infection and Tuberculosis Coprevalence
Source: J Infect Dis. 2017 Jul 20;216(6):632–40. doi: 10.1093/infdis/jix337 (PMC5815623; doi:10.1093/infdis/jix337)
Supplement: Supplementary_Table_1 [file jix337_suppl_supplementary_table_1.docx]

**Supplementary Table 1 Extended phenotypic drug sensitivity testing**

| **Critical concentration µg/ml used in sensititre MycoTB plates** | | | | | | | | | | | |
| --- | --- | --- | --- | --- | --- | --- | --- | --- | --- | --- | --- |
| **Drugs** | | | | | | | | | | | |
| **INH** | **RMP** | **OFL** | **MXF** | **AM** | **SM** | **RBU** | **PAS** | **ETH** | **CYC** | **KAN** | **EMB** |
| **0.2/1** | **1** | **2** | **2** | **5** | **2/10** | **0.5** | **2** | **5** | **25** | **5** | **5/10** |

Abbreviations:

INH isoniazid, RIF rifampicin, OFL ofloxacin, MXF moxifloxacin, AM amikacin, SM streptomycin, RBU rifabutin PAS para-aminosalicylic acid, ETH ethionamide, CYC cyclserine, KAN kanamycin, EMB ethambutol
