# Supplementary material for: Low Frequency of Acquired Isoniazid and Rifampicin Resistance in Rifampicin-Susceptible Pulmonary Tuberculosis in a Setting of High HIV-1 Infection and Tuberculosis Coprevalence
Source: J Infect Dis. 2017 Jul 20;216(6):632–40. doi: 10.1093/infdis/jix337 (PMC5815623; doi:10.1093/infdis/jix337)
Supplement: Supplementary_Table_2 [file jix337_suppl_supplementary_table_2.docx]

Supplementary table 2 Ascertainment of acquired rifampicin and isoniazid resistance during treatment

| Time point | Overall study cohort n=306 | |
| --- | --- | --- |
|  | Screening for acquired rifampicin and acquired isoniazid resistance via MTBDR*plus* on positive culture, n | Outcome of participants who did not produce sputum for culture |
| 2 months | 287 | Died n=2  Lost to follow up n=8  Assessment of smear conversion only n=9 |
| 5-6 months | 240 | Treatment completers/smear converters n=40*  Defaulters n=10  Treatment outcome unknown n=10 |

* These treatment completers were classified as not acquiring drugs resistance if they successfully completed treatment and did not re-present with drug resistant TB recurrence during the study follow up period.
