# Supplementary material for: Low Frequency of Acquired Isoniazid and Rifampicin Resistance in Rifampicin-Susceptible Pulmonary Tuberculosis in a Setting of High HIV-1 Infection and Tuberculosis Coprevalence
Source: J Infect Dis. 2017 Jul 20;216(6):632–40. doi: 10.1093/infdis/jix337 (PMC5815623; doi:10.1093/infdis/jix337)
Supplement: Supplementary_Table_3 [file jix337_suppl_supplementary_table_3.docx]

**Supplementary Table 3 Treatment outcomes by HIV-1 serostatus**

| Outcome | Overall | HIV-1 co-infected | HIV-1 uninfected |
| --- | --- | --- | --- |
| 2-month culture conversion* | 228/289 (79%) | 149/180 (82%) | 79/107 (74%) |
| Culture conversion/ treatment completion at 5-6 months** | 270/296 (92%) | 159/186 (89%) | 101/110 (94%) |
| Failure** Default | 10/296 (3%)  10/296 (3%) | 7/186 (4%) 7/186 (4%) | 3/110 (3%) 3/110 (3%) |
| Died during treatment** | 6/296 (2%) | 6/186 (3%) | 0/110 (0%) |
| Recurrence | 9/270 (3%) | 7/166 (4%) | 3/104 (3%) |
| Died post treatment | 5/270 (1%) | 5/166 (1%) | 0/104 (0%) |

*17 participants did not produce sputum at 2 months.

**10 participants had unknown outcome and were not included in the denominator
