# Supplementary figures and images for: Low Frequency of Acquired Isoniazid and Rifampicin Resistance in Rifampicin-Susceptible Pulmonary Tuberculosis in a Setting of High HIV-1 Infection and Tuberculosis Coprevalence
Source: J Infect Dis. 2017 Jul 20;216(6):632–40. doi: 10.1093/infdis/jix337 (PMC5815623; doi:10.1093/infdis/jix337)

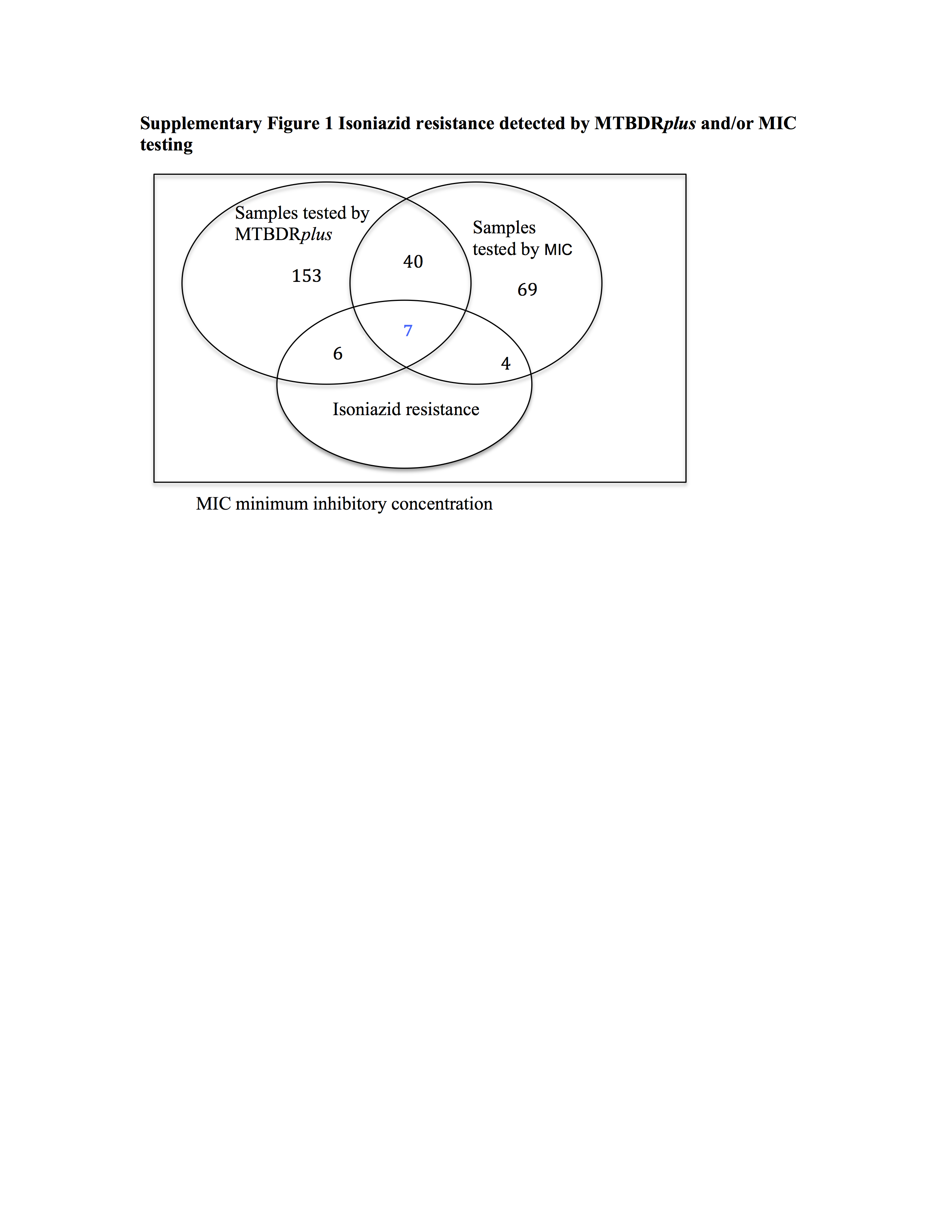

Supplement: Supplementary_Figure_1 [file jix337_suppl_supplementary_figure_1.png]
