# Supplementary material for: Low Frequency of Acquired Isoniazid and Rifampicin Resistance in Rifampicin-Susceptible Pulmonary Tuberculosis in a Setting of High HIV-1 Infection and Tuberculosis Coprevalence
Source: J Infect Dis. 2017 Jul 20;216(6):632–40. doi: 10.1093/infdis/jix337 (PMC5815623; doi:10.1093/infdis/jix337)
Supplement: Supplementary_methods [file jix337_suppl_supplementary_methods.docx]

Supplementary methods

**Minimum Inhibitory Concentration (MIC) determination**

A MGIT 960 system with EpiCenter software was used to determine MIC according to the 1% proportion method [1]. Bacterial suspensions were made from frozen stock (-80^o^C) in MGIT 960 medium grown at 37°C. Inocula were then prepared from MGIT subcultures 2 days after the tubes flagged positive. INH and RIF were purchased from Sigma Aldrich, South Africa while the BACTEC MGIT 960 PZA Kit (Becton Dickinson Biosciences, Sparks, USA) was used for PZA. INH and PZA was dissolved in sterile distilled water (SDW) and RIF in dimethyl sulfoxide (DMSO) with subsequent dilution in SDW. Stock solutions of the drugs were prepared at concentrations 84 times higher than the highest test concentrations required. These solutions were filter sterilized and stored at -80°C in small aliquots for not longer than 6 months. Serial 2-fold dilutions were made in SDW to obtain working solutions ranging from 21 – 168 mg/L; 2.54 – 84 mg/L and 2118 – 8400 mg/L for INH, RIF and PZA, respectively. From each drug dilution, 0.1 ml quantities of INH and RIF were transferred to standard MGIT tubes containing 7.0 ml modified Middlebrook 7H9 broth base (pH 6.8) supplemented with 0.8 ml oleic acid-albumin-dextrose-catalase (OADC). The procedure for PZA was similar, apart from using MGIT 960 PZA medium (Becton Dickinson Biosciences, Sparks) at a reduced pH of 5.9. The tubes were then inoculated with 0.5 ml of the test organisms to give final 2-fold drug concentrations ranging from 0.25 – 2 mg/L, 0.03 – 1.0 mg/L and 25 – 100 mg/L for INH, RIF and PZA, respectively. A drug-free 1:100 diluted inoculum (1:10 for PZA) was included to represent the 1% critical proportion which is used to differentiate between susceptible and resistant bacilli. The interpretation of the results were based on a threshold growth unit (GU) reading of 400 by the drug-free control. Drug containing tubes with GU readings of ≥100 at the time when the drug-free control reached a value of 400 were considered resistant and those with values <100 as susceptible. The MIC was therefore defined as the lowest drug concentration that inhibits growth of more than 99% of the bacterial population. Critical concentrations of 0.1 mg/L, 1.0 mg/L and 100 mg/L were used to define resistance to INH, RIF and PZA, respectively.

1. Cambau E, Viveiros M, Machado D, et al. Revisiting susceptibility testing in MDR-TB by a standardized quantitative phenotypic assessment in a European multicentre study. J Antimicrob Chemother **2015**; 70:686-96.
